# Synovium-Derived and Bone-Derived Mesenchymal Stem/Stromal Cells from Early OA Patients Show Comparable In Vitro Properties to Those of Non-OA Patients

**DOI:** 10.3390/cells13151238

**Published:** 2024-07-23

**Authors:** Janja Zupan, Klemen Stražar

**Affiliations:** 1 Department of Clinical Biochemistry, Faculty of Pharmacy, University of Ljubljana, Askerceva 7, 1000 Ljubljana, Slovenia; janja.zupan@ffa.uni-lj.si; 2 Department of Orthopaedic Surgery, University Medical Centre Ljubljana, Zaloska 9, 1000 Ljubljana, Slovenia; 3 Faculty of Medicine, University of Ljubljana, Vrazov trg 2, 1000 Ljubljana, Slovenia

**Keywords:** mesenchymal stem/stromal cells, early OA, synovium, podoplanin, CD146

## Abstract

Degenerative disorders like osteoarthritis (OA) might impair the ability of tissue-resident mesenchymal stem/stromal cells (MSCs) for tissue regeneration. As primary cells with MSC-like properties are exploited for patient-derived stem cell therapies, a detailed evaluation of their in vitro properties is needed. Here, we aimed to compare synovium-derived and bone-derived MSCs in early hip OA with those of patients without OA (non-OA). Tissues from three synovial sites of the hip (paralabral synovium, cotyloid fossa, inner surface of peripheral capsule) were collected along with peripheral trabecular bone from 16 patients undergoing hip arthroscopy (8 early OA and 8 non-OA patients). Primary cells isolated from tissues were compared using detailed in vitro analyses. Gene expression profiling was performed for the skeletal stem cell markers podoplanin (*PDPN*), *CD73*, *CD164* and *CD146* as well as for immune-related molecules to assess their immunomodulatory potential. Synovium-derived and bone-derived MSCs from early OA patients showed comparable clonogenicity, cumulative population doublings, osteogenic, adipogenic and chondrogenic potential, and immunophenotype to those of non-OA patients. High *PDPN*/low *CD146* profile (reminiscent of skeletal stem cells) was identified mainly for non-OA MSCs, while low *PDPN*/high *CD146* mainly defined early OA MSCs. These data suggest that MSCs from early OA patients are not affected by degenerative changes in the hip. Moreover, the synovium represents an alternative source of MSCs for patient-derived stem cell therapies, which is comparable to bone. The expression profile reminiscent of skeletal stem cells suggests the combination of low *PDPN* and high *CD146* as potential biomarkers in early OA.

## 1. Introduction

The synovial joints are reservoirs of endogenous mesenchymal stem/stromal cells (MSCs) with regenerative capabilities [1]. In current clinical orthopedics, bone marrow and adipose tissue are the most common sources for patient-derived cell therapies [2,3,4]. However, previous studies that have investigated bone-derived MSCs from patients with advanced osteoarthritis (OA) have shown that they have reduced chondrogenic, adipogenic and osteogenic activities in vitro in comparison to those of healthy donors [5,6]. A similar study showed the accumulation of CD271-positive cells in bone adjacent to cartilage defects and areas of osteochondral angiogenesis, and these MSCs showed less proliferation and mineralization [7]. Another study identified MSCs with an OA phenotype in the cartilage of patients undergoing hip replacement surgery [8]. However, there remains a lack of evidence as to whether changes in progenitor cells and MSCs are associated with aging and/or OA [9].

In addition to the OA that affects the aging population, there has also been the suggestion that stem cell exhaustion might arise as a consequence of advanced age [10]. As OA has been recognized as a disorder of the whole joint [11], other tissues in close proximity to synovial joints might be impaired in the same way as bone and bone marrow, in terms of their endogenous reparative capacities.

Our previous data have shown reduced osteogenic and chondrogenic potential of bone-derived MSCs from patients with primary OA of the hip, in comparison with OA patients with dysplasia, which suggests that the pathology of primary OA is accompanied by ‘exhaustion’ of bone-derived MSCs [12]. Moreover, our comprehensive analysis of skeletal-muscle-derived and bone-derived MSCs showed exhaustion of these cells in OA and fragility fracture patients [5]. A recent study identified aging as associated with progressive loss of bone-marrow-resident skeletal stem cells [13], which were previously identified as positive for podoplanin (PDPN), CD73 and CD164, and negative for CD146 [14]. Moreover, these authors also showed age-related diminished chondrogenesis of these cells in the joints of both mice and humans [13]. However, the same study showed that these stem cells can be activated by local microfracture surgery, and expanded by local delivery of bone morphogenetic protein 2 (BMP2) and a soluble vascular endothelial growth factor receptor antagonist (sVEGFR1), to generate cartilage for treatment of localized chondral disease in OA [13].

Synovium-derived MSCs represent a promising alternative source to bone-marrow-derived and adipose-tissue-derived MSCs for stem cell therapies, which show the greatest chondrogenic potential for healthy articular cartilage growth with minimal hypertrophic differentiation [15,16,17,18,19,20,21]. Following joint injury, synovium hyperplasia occurs as a result of the endogenous expansion of MSCs that can regenerate cartilage [22] or even form joint-like structures [1] as shown in mouse models of OA. However, in humans, the potential use of synovium-derived MSCs has been far less recognized compared to bone-marrow-derived and adipose tissue-derived MSCs [2,3].

Human synovium-derived MSCs have so far shown promising in vitro potential in various donors and patients [19,23,24,25]. In their evaluation of yields and standard in vitro tests for expandability, differentiation potential and epitope profiles, Sakaguchi et al. demonstrated superior chondrogenesis and adipogenesis of synovium-derived MSCs in comparison to MSCs from bone marrow, periosteum, adipose tissue and skeletal muscle of patients during anterior cruciate ligament reconstruction surgery [25]. Kohno et al. reported that yields, surface markers and chondrogenic potential of primary synovium-derived MSCs in patients with rheumatoid arthritis were comparable to those with OA [23]. Murata et al. showed that synovium-derived cells from the cotyloid fossa of patients with femoroacetabular impingement syndrome had higher proliferation and differentiation potential than those from the paralabral synovium, which suggested that MSCs from the cotyloid fossa might indeed be strongly considered for stem cell therapy [19]. However, clinical studies that use human synovium MSCs are still in their infancy, although Sekiya et al. showed that transplantation of synovium-derived MSCs was effective in terms of magnetic resonance imaging, qualitative histology and Lysholm scores in patients with symptomatic single cartilage lesion of the femoral condyle [26].

Most of these studies have evaluated patients with advanced joint disorders, so much less is known about MSCs in patients with early degenerative changes in their joints. Another hurdle is that the identity or immunophenotype of human synovium-derived MSCs remains obscure. In contrast, the immunophenotype of human bone-marrow-resident skeletal stem cells was not long ago identified as positive for podoplanin (PDPN), CD73 and CD164, and negative for CD146 [14].

The aim of the present study was to perform detailed in vitro analyses of primary synovium-derived and bone-derived MSCs in patients who showed early degenerative changes in the hip (early OA) and in those with no such changes apparent (non-OA). We hypothesized that synovium-derived and bone-derived MSCs are comparable between early OA and non-OA patients, in terms of colony formation, growth kinetics, multipotency, immunophenotype and gene expression profiles of skeletal stem cell markers and immune-related molecules. To test this hypothesis, we harvested tissues from three different synovial sites, i.e., the paralabral synovium, cotyloid fossa and inner surface of the peripheral capsule, and from the peripheral bone of 16 patients undergoing hip arthroscopy. Early degenerative changes (early OA) of the hip were observed in eight of these patients, while the other eight patients showed no hip degeneration (non-OA). We isolated and cultured primary cells from these tissues, and performed detailed characterization of their in vitro MSC-like properties. These data show no differences in the biological properties of the MSCs between the early OA and non-OA patients. Interestingly, gene expression profiling identified a cluster of MSCs with high *PDPN* and low *CD146* gene expression that were derived mainly from non-OA patients, similar to the skeletal stem cell phenotype. In contrast, MSCs from early OA patients tended to express low *PDPN* and high *CD146*.

## 2. Materials and Methods

### 2.1. Patient Inclusion and Tissue Sampling

Patients with various indications for hip arthroscopy were included in the study at the Department of Orthopedic Surgery, University Medical Centre Ljubljana (Ljubljana, Slovenia). The exclusion criteria included a history of inflammatory arthritis, metastatic cancer and other disorders directly affecting bone, such as osteonecrosis. Approval for this study was obtained from the National Medical Ethics Committee of the Republic of Slovenia (reference number: 0120-499/2020/7). Written informed consent was obtained from all of the patients included in the study.

**Figure 1:**
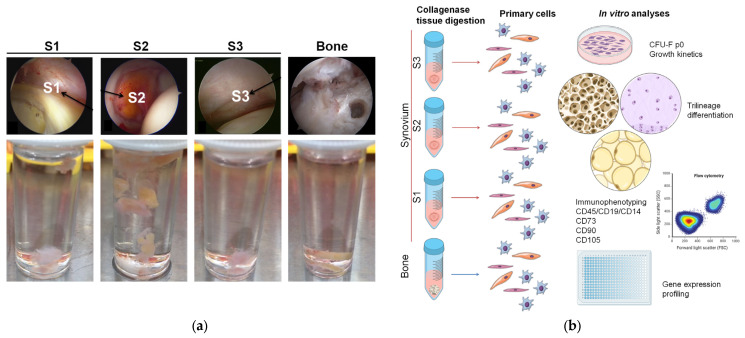
The protocol of the study. (**a**) Representative arthroscopic (upper row) and macroscopic (lower row) images of synovium tissue harvested from three different sites in the hip and trabecular bone biopsies for primary cell isolation. (**b**) Scheme of the primary cell isolation and analyses. S1, paralabral synovium, S2, cotyloid fossa, S3, peripheral inner surface of the joint capsule, CFU-F p0, colony-forming unit fibroblast at passage 0.

The synovium and trabecular bone were sampled from all of the patients included in the study. Synovium was harvested at three different sites: the paralabral synovium in the perilabral sulcus (S1), the cotyloid fossa (S2), and the inner surface of the peripheral joint capsule (S3), as illustrated in Figure 1a. A cylindrical section of trabecular bone with bone marrow was sampled from the anterior head–neck junction. All of the tissues were placed separately in a growth medium of low-glucose Dulbecco-modified Eagle’s medium (DMEM; Biowest) supplemented with 1% glutamine and 2% penicillin and streptomycin (all Biowest), and 10% fetal bovine serum (Gibco), and stored at 4 °C until cell isolation as summarized in Figure 1b.

### 2.2. Primary Cell Isolation

Primary cells were separately isolated from each of the 64 tissue samples within 2 to 70 h post-arthroscopy. Each of the tissues was transferred to sterile phosphate-buffered saline in a cell culture laboratory and weighed prior to cell isolation. For the synovium and bone cell isolation, previously published protocols were followed, with slight modifications [24,27]. Briefly, each sample from each of the four tissues was digested separately in 1 mg/mL collagenase solution (>0.15 U/mg; Roche, Basel, Switzerland) at 37 °C for 3 h. The digested tissues were filtered through a 70-µm nylon strainer (Corning, Corning, NY, USA). An additional 10 mL of fresh medium was added to the bone remnants, which were then vortexed for 10 s. This was repeated twice more. The cumulative suspensions (approximately 30 mL) were centrifuged at 300× *g* for 10 min.

The cell pellets from each of the individual tissue samples were resuspended in fresh medium, and the cells were plated for the colony-forming unit fibroblast (CFU-F) assays using StemMACS MSC Expansion Media Kit XF (human; Miltenyi Biotec, Bergisch Gladbach, Germany). The cells were incubated at 37 °C under 5% humidified CO_2_ and O_2_.

### 2.3. Colony-Forming Unit Fibroblast Assay

The colony-forming unit fibroblast (CFU-F) assays were performed at passage 0 as described previously, with slight modifications [5,12,28]. Briefly, freshly isolated primary cells were plated as six replicates in six-well plates. Once the colonies were established (i.e., after 10 to 27 days, ≥5 population doublings, or 32 cells per colony), they were counted in the wells under a digital inverted phase-contrast microscope (Evos XL; Life Technologies, Carlsbad, CA, USA). After counting the colonies, the wells were trypsinized and the viable cells were counted using a Neubauer chamber. The colony numbers from the CFU-F at p0 were calculated as proportions (%) of the counted colonies per cells counted.

### 2.4. Cell Expansion

Following trypsinization at p0, the cells were further culture expanded, from 5000 cells/cm^2^. The culture expansion was carried out in a growth medium of low-glucose DMEM (Biowest, Nuaillé, France) supplemented with 1% glutamine and 2% penicillin and streptomycin (all Biowest), and 10% fetal bovine serum (Gibco, Billings, MT, USA). Cumulative population doublings were calculated as 3.32 × (logN2 − logN1), where N1 is the number of seeded cells at each passage, and N2 is the number of counted cells at each passage, as described previously [12].

### 2.5. Multilineage Differentiation

Primary cells were seeded as four replicates in 24-well plates at 50,000 cells/well for osteogenesis at passage 2 and for adipogenesis at passage 3. Two replicates (i.e., treated, control) were used for histological assessment, and two for RNA isolation. The treated replicate received either adipogenic medium (i.e., growth medium supplemented with 500 nM dexamethasone, 10 µM indomethacin, 50 µM isobutylmethyl xanthine, 10 µg/mL insulin [all Sigma, Burlington, MA, USA]) or osteogenic medium (i.e., growth medium supplemented with 5 mM β-glycerophosphate, 100 nM dexamethasone, 50 mg/mL ascorbic acid-2-phosphate [all Sigma]). The control replicates received growth medium without these adipogenic or osteogenic supplements. The treatments were continued for 21 days, with medium changes every 2 to 3 days.

After 21 days, the adipogenic cultures were stained with Oil Red O (Sigma) and the osteogenic cultures with 2% Alizarin Red S (Sigma). After staining, the wells were imaged using a digital inverted bright-field and phase-contrast microscope (Evos XL; Life Technologies). The numbers of adipocytes were counted using the ImageJ software (version 1.54g) [29]. The adipogenic potential was calculated as the number of Oil-Red-O-positive adipocytes per the number of seeded cells. The Alizarin Red S was subsequently extracted using 5% sodium dodecyl sulphate in 0.5 M hydrochloric acid. The Alizarin Red S standard curve was generated using serial dilutions and measured along with the cell extracts at 405 nm, using a microplate reader (Safire II; Tecan, Männedorf, Switzerland). The osteogenic potential was calculated as the concentration of Alizarin Red S (mg/mL).

For chondrogenesis, cell pellets were formed from cells at passage 1 as two replicates of 150,000 cells that were suspended in chondrogenic medium (i.e., high-glucose DMEM [Biowest], 100 nM dexamethasone [Sigma], 1% insulin-transferrin-selenium [Sigma], 50 mg/mL ascorbic acid-2-phosphate [Sigma], 1% penicillin/streptomycin [Biowest]). The treated pellets received 10 ng/mL TGF-ß1 (ThermoFisher Scientific, Waltham, MA, USA), and the controls received medium without TGF-ß1. The treatments were continued for 21 days, with medium renewal every 2 to 3 days. After 21 days, the pellets were fixed in 5% neutral buffered formalin and kept in 70% ethanol until they were embedded in paraffin and cut into 5-µm-thick sections. The sections were stained with toluidine blue (Sigma) and immunostained for collagen type II (Col2). For Col2 staining, a goat anti-Col2 antibody conjugated with Alexa Fluor 488 (catalogue N° 1320-30; SouthernBiotech, Birmingham, AL, USA) was used at a 1:50 dilution. The slides were mounted with Prolong Gold Antifade Reagent with DAPI (Life Technologies), and imaged using a digital inverted fluorescent microscope (Evos FL; Life Technologies). The chondrogenic potential was determined as the proportion (%) of the chondrogenic pellets stained positive for toluidine blue or Col2. Sections stained with toluidine blue were also analyzed using the Bern score [30], and the diameter of the chondrogenic pellet was measured using the ImageJ software [29]. The images were blindly evaluated by two independent observers.

### 2.6. Immunophenotyping

Flow cytometry was performed on culture-expanded MSCs at passage 3 as described previously [5,12,28]. Briefly, the following antibodies were used: anti-CD105 MEM-226 FITC (ThermoFisher Scientific), anti-CD90 DG3 FITC, anti-CD73 AD2 APC (Miltenyi Biotec) and viability dye eFluor 780 (ThermoFisher Scientific). Flow cytometry was performed using an Attune NxT instrument (ThermoFisher Scientific).

### 2.7. Gene Expression

Total RNA was isolated from the cells subjected to adipogenesis and osteogenesis, as well as from undifferentiated cells during culture expansion at passage 3. Total RNA was extracted using qGOLD Total RNA kits (VWR) and the cDNA was synthesized using High-Capacity cDNA Reverse Transcription kits (ThermoFisher Scientific). Gene expression measurements were performed according to the ‘Minimum Information for Publication of Quantitative Real-time PCR Experiments’ guidelines [31].

Quantitative PCR was performed using 5× HOT FIREPol EvaGreen qPCR Supermix (Solis BioDyne OÜ, Tartu, Estonia), according to the manufacturer protocol. The sequences of the primers (Macrogen, Sigma-Aldrich) used to measure osteogenesis and adipogenesis-related genes as well as inflammation and immunomodulation-related genes were provided in our [5,12,28,32,33], and other, previous studies [34,35,36,37]. All of the PCR amplifications were performed in triplicate in a 15 μL reaction volume in a PCR machine (LightCycler 480 Instrument II; Roche). Gene expression data were obtained using a standard curve and the second derivative maximum method (LightCycler 480 software, v. 1.5.0). All of the data were normalized to glyceraldehyde-3-phosphate dehydrogenase.

### 2.8. Statistical Analysis

The normality of the data distribution was tested with a Shapiro–Wilk test. Patient characteristics (e.g., age, body mass index) between the early OA and non-OA groups were compared with Mann–Whitney tests. To compare the data between the four tissue groups between the early OA and non-OA groups, two-way ANOVA with Bonferroni corrections for multiple testing was used. Due to the limited availability of the samples derived from S1, and to increase the power of the statistical analysis, samples from all three synovial groups were combined into a single group (S1 + S2 + S3). Differences in the characteristics of the primary cells from synovium and bone were compared between the early OA and non-OA patients using the general linear model, and Bonferroni post hoc tests. As age showed a significant difference between the tested groups of patients, age was used as a covariate in all analyses. Hence, the differences reported in this study are the result of early OA and are not age-dependent. The statistical analyses were performed using IBM SPSS Statistics, version 29 (IBM Corp., Armonk, NY, USA) and GraphPad Prism, version 8 (GraphPad Software, La Jolla, CA, USA; www.graphpad.com). *p* < 0.05 was considered statistically significant. Heat maps were generated from the normalized gene expression data using the online Heatmapper software [38]. The figures were created using BioRender (www.BioRender.com) and Mind the Graph (www.mindthegraph.com (accessed on 14 June 2024)).

## 3. Results

### 3.1. Patients Characteristics

We included 16 patients in our study. The full individual patient clinical characteristics and arthroscopic findings are given in Table 1. In eight of the patients, early OA (i.e., classified as grade I according to Tönnis [39]) was diagnosed preoperatively by clinical examination, plain radiographs and magnetic resonance arthrography, and was further confirmed during arthroscopy (i.e., the early OA patients). The other eight patients showed no hip degeneration changes and served as the non-OA group.

There was a significant difference in median age between the early OA and non-OA patients (47.5 vs. 23.5 years; *p* = 0.0012, Mann–Whitney test). There were no significant differences in sex (female/male ratio, 6/2 for both groups) or median body mass index (24.7 vs. 22.9 kg/m^2^; *p* = 0.1304, Mann–Whitney test) between the two groups.

### 3.2. Tissue Samples and Isolation Efficacy of the Primary Cells

**Figure 2:**
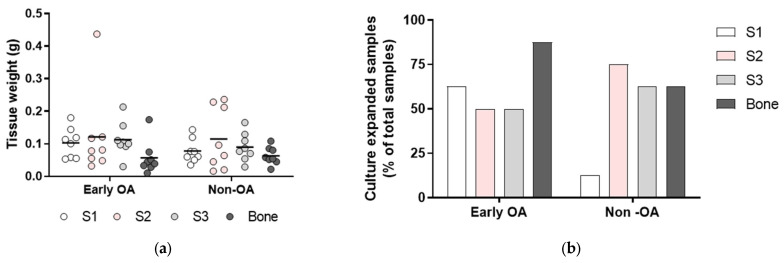
Tissue weight and isolation efficacy. (**a**) The weight of the tissue biopsies used for cell isolation. Individual samples and means are shown. There were no differences in tissue weight between the patient groups (two-way ANOVA with Bonferroni multiple comparison tests). (**b**) Shown is the isolation efficacy in each tissue (N = 16 donors). S1, paralabral synovium, S2, cotyloid fossa, S3, peripheral inner surface of the joint capsule.

The mean amounts of biopsies harvested for cell isolation were similar for all four tissues (S1-S3; bone) between the two groups of patients (early OA, non-OA; respectively): S1, 0.103 g, 0.078 g; S2, 0.121 g, 0.115 g; S3, 0.113 g, 0.090 g; bone, 0.057 g, 0.063 g (Figure 2a). In general, these resulted in similar proportions of successfully culture-expanded samples of primary cells between the two groups of eight patients (early OA/non-OA; respectively): S1, 5/1; S2 4/6; S3 4/5; bone 7/5 (Figure 2b). Of note, for S1, a higher proportion of samples was isolated for the early OA patients (five from eight) in comparison with the non-OA patients (one from eight). Since only one sample was culture expanded from paralabral synovium, i.e., S1 in the group of non-OA patients, the data from all three synovium groups, namely S1, S2 and S3, were combined into a single group for further comparison of synovium and bone-derived primary cells between both patient groups.

### 3.3. Culture Expansion and Growth Kinetics

**Figure 3:**
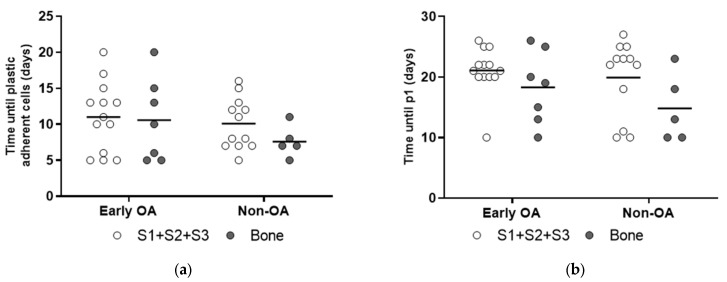
Time until primary culture establishment. (**a**) No differences in time until plastic adherent cells were first observed were found for synovium and bone-derived cells between both patient groups (*p* = 0.521, general linear model with Bonferroni post hoc). (**b**) No differences in time until passage 1 (p1) were shown for the synovium and bone-derived cells between both patient groups (*p* = 0.521, general linear model with Bonferroni post hoc). Individual samples and means are shown. S1 + S2 + S3 presents a group of combined data from S1, paralabral synovium, S2, cotyloid fossa and S3, peripheral inner surface of the joint capsule.

**Figure 4:**
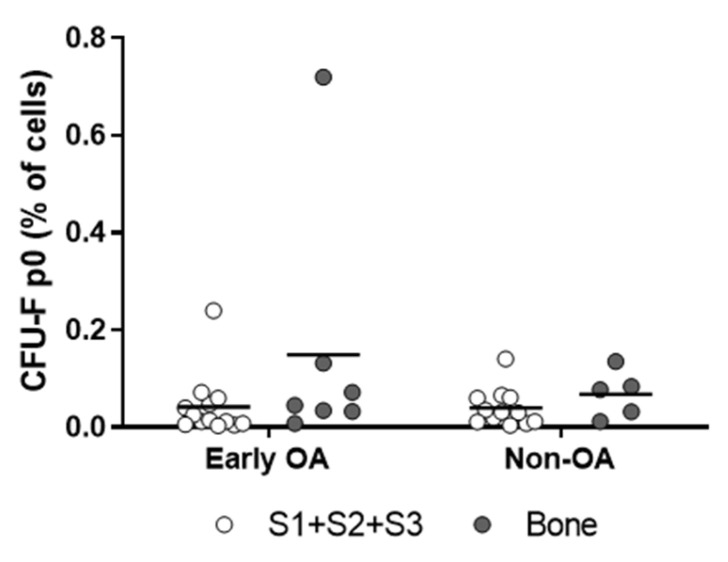
Results of colony forming unit fibroblast (CFU-F) activity of the freshly seeded cells at p0. Individual samples and means are shown. There were no differences in CFU-F of the synovium and bone-derived cells between both patient groups (*p* = 0.369, general linear model with Bonferroni post hoc). S1 + S2 + S3 presents a group of combined data from S1, paralabral synovium, S2, cotyloid fossa and S3, peripheral inner surface of the joint capsule. The representative images of colonies formed for each sample are included in Supplementary Figure S1.

Plastic-adherent cells were observed at similar times for all of these tissues from both groups of patients (Figure 3a). There were also no differences in the times until the first passage (p1) and the clonogenicities (Figure 3b and Figure 4). The representative images of the colonies formed at p0 are shown in Supplementary Figure S1.

**Figure 5:**
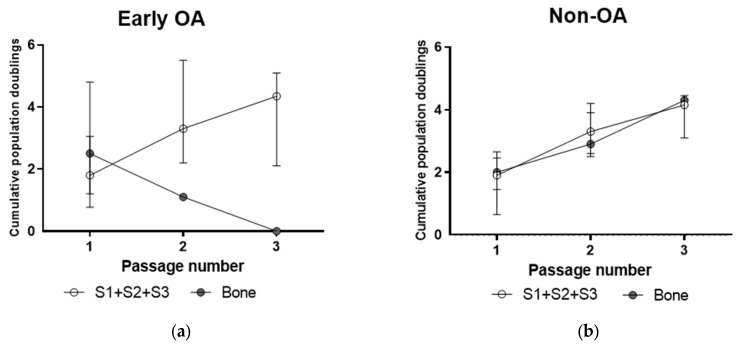
Growth kinetics of the primary cells from both patient groups. (**a**) Cumulative population doublings of cells from early OA patients. (**b**) Cumulative population doublings of cells from non-OA patients. Shown are medians with interquartile range. No significant differences were observed between cells from synovium and bone (general linear model with Bonferroni post hoc). S1 + S2 + S3 presents a group of combined data from S1, paralabral synovium, S2, cotyloid fossa and S3, peripheral inner surface of the joint capsule.

The primary cells from both tissues showed similar growth kinetics, as shown by the cumulative population doublings between the early OA samples (Figure 5a) and non-OA samples (Figure 5b) with primary cells from early OA showing broad donor-to-donor variability (Figure 5a).

### 3.4. Multilineage Differentiation

**Figure 6:**
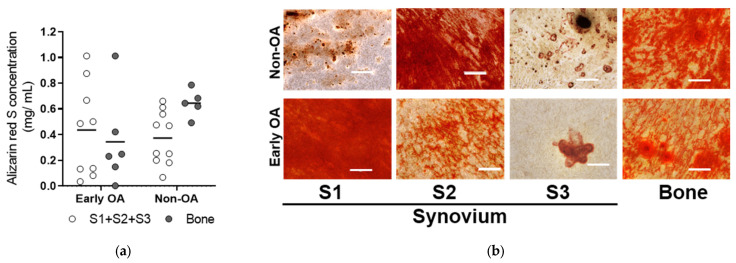
Alizarin Red S histochemistry to assess the osteogenic potential of the primary cells. (**a**) Osteogenesis was assessed at passage 2 using Alizarin Red S quantification. No differences for synovium and bone-derived cells between both groups of patients (general linear model with Bonferroni post hoc) were shown. Individual samples and means are shown. S1 + S2 + S3 presents a group of combined data from S1, paralabral synovium, S2, cotyloid fossa and S3, peripheral inner surface of the joint capsule. (**b**) Representative images for each tissue in early OA and non-OA patients are shown. Scale bars: 200 µm.

**Figure 7:**
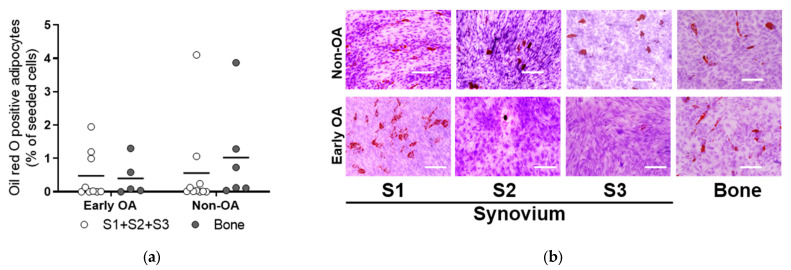
Oil Red O staining to assess the adipogenic potential of the primary cells. (**a**) Adipogenesis was assessed at passage 3 using quantification of Oil Red O positive adipocytes. No differences for synovium and bone-derived cells between both groups of patients (general linear model with Bonferroni post hoc) were shown. Individual samples and means are shown. S1 + S2 + S3 presents a group of combined data from S1, paralabral synovium, S2, cotyloid fossa and S3, peripheral inner surface of the joint capsule. (**b**) Representative images for each tissue in early OA and non-OA patients are shown. Scale bars: 200 µm.

**Figure 8:**
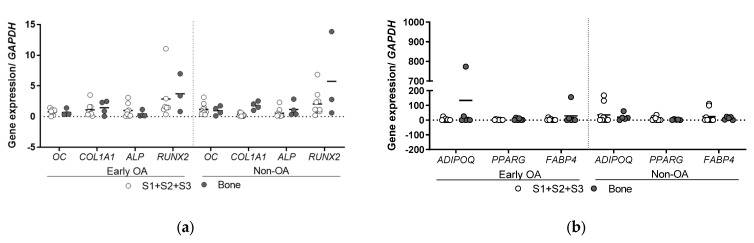
Gene expression of osteogenic and adipogenic-related genes. (**a**) Osteogenesis was also assessed by gene expression measurement of osteogenic genes, osteocalcin (*OC*), collagen type 1 alpha 1 (*COL1A1*), alkaline phosphatase (*ALP*) and runt-related transcription factor 2 (*RUNX2*). (**b**) Adipogenesis was also assessed by gene expression measurement of adipogenic genes, adiponectin (*ADIPOQ*), peroxisome proliferator-activated receptor gamma (*PPARG*) and fatty acid binding protein 4 (*FABP4*). Individual samples and means are shown. No differences for synovium and bone-derived cells between both groups of patients (general linear model with Bonferroni post hoc) were observed for any of the genes. S1 + S2 + S3 presents a group of combined data from S1, paralabral synovium, S2, cotyloid fossa and S3, peripheral inner surface of the joint capsule.

**Figure 9:**
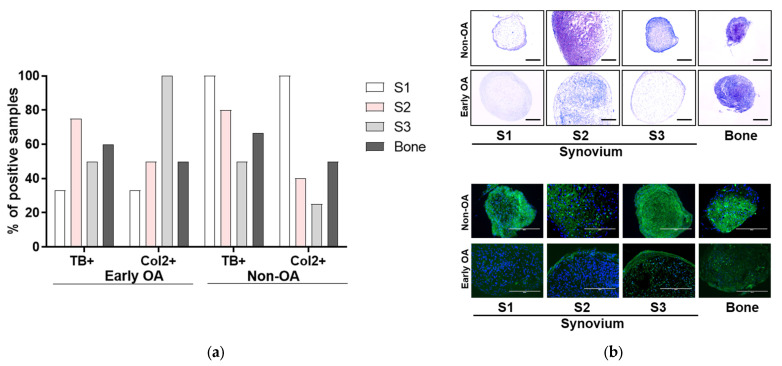
Toluidine blue staining and Col2 immunofluorescence staining to assess the chondrogenic differentiation of the primary cells at passage 1. (**a**) Quantification of toluidine blue (TB) and collagen type II (Col2) positive chondrogenic pellets. (**b**) Representative images of toluidine blue (upper row) and collagen type II (lower row) stained chondrogenic pellets for each tissue in early OA and non-OA patients are shown. Scale bars: 200 µm.

**Figure 10:**
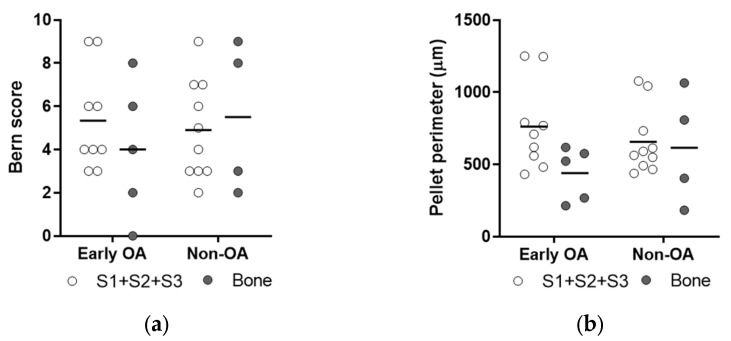
Quantification of the chondrogenic potential of primary cells. (**a**) Bern score was used to evaluate the in vitro generated neocartilage in chondrogenic pellets stained with toluidine blue. No differences for synovium and bone-derived cells between both groups of patients (general linear model with Bonferroni post hoc) were shown. Individual samples and means are shown. S1 + S2 + S3 presents a group of combined data from S1, paralabral synovium, S2, cotyloid fossa and S3, peripheral inner surface of the joint capsule. (**b**) Perimeters of the chondrogenic pellets stained with toluidine blue were measured. The results showed no differences for synovium and bone-derived cells between both groups of patients (general linear model with Bonferroni post hoc).

To determine whether these culture-expanded synovium-derived and bone-derived cells had features of MSCs in vitro according to the recommendations of the International Society for Cellular Therapy (ISCT) [40], their osteogenic, adipogenic and chondrogenic potentials were evaluated (Figure 6, Figure 7, Figure 8, Figure 9 and Figure 10). These MSCs showed variable levels of osteogenic (Figure 6) and adipogenic (Figure 7) differentiation, as seen by histological staining and expression of lineage-specific genes (Figure 8), with no significant differences between the same tissue sources from both patient groups. The means of Alizarin Red S concentration (mg/mL) were similar for all four tissues (S1–S3; bone) between the two groups of patients (early OA, non-OA; respectively): S1, 0.587, 0.255; S2, 0.475, 0.405; S3, 0.135, 0.364; bone, 0.344, 0.645 (Figure 6). The means of Oil Red O (%) were similar for all four tissues (S1-S3; bone) between the two groups of patients (early OA, non-OA; respectively): S1, 0.981, 4.102; S2, 0.303, 0.060; S3, 0.079, 0.306; bone, 0.402, 1.026 (Figure 7).

The chondrogenic potentials were also not significantly different between the tissue sources, with similar rates of toluidine blue and Col2-positive chondrogenic pellets (Figure 9), and similar Bern scores and pellet perimeters (Figure 10).

### 3.5. Immunophenotyping

**Figure 11:**
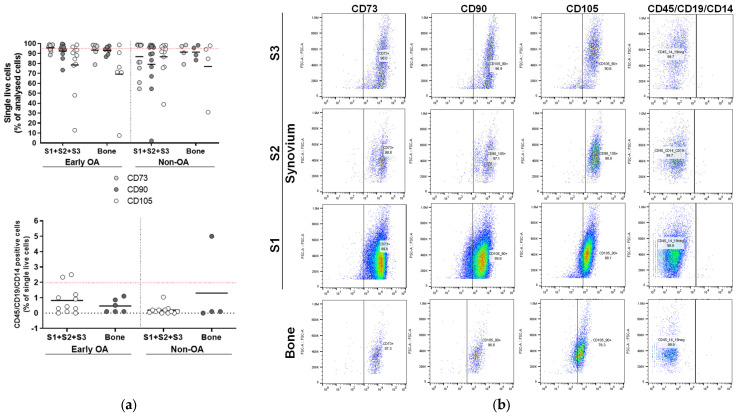
Immunophenotype of the in vitro expanded primary cells at passage 3. (**a**) The percentage of the cells expressing the positive markers CD73, CD90 and CD105 (upper row), and negative markers CD45/CD19/CD14 (lower row). Red dash lines represents the recommendation of the International Society for Cellular Therapy [40] criteria suggesting that positive markers are expressed by more than 95% of all of the cells and negative markers are expressed by less than 2% of all of the cells. No differences for synovium and bone-derived cells between both groups of patients (general linear model with Bonferroni post hoc) were found. Individual samples and means are shown. S1 + S2 + S3 presents a group of combined data from S1, paralabral synovium, S2, cotyloid fossa and S3, peripheral inner surface of the joint capsule. (**b**) Shown are representative dot plots for each tissue type independent of patient group. S1, paralabral synovium, S2, cotyloid fossa, S3, peripheral inner surface of the joint capsule.

To further determine whether these culture-expanded primary cells had MSC-like features in vitro according to the recommendations of the ISCT [40] their immunophenotypes were determined (Figure 11). The MSCs from both of these patient groups showed similar immunophenotypes, with high expression of the positive markers CD73, CD90 and CD105, and low expression of the negative markers CD45, CD14 and CD19 (Figure 11a).

However, the criteria set by the ISCT that require >95% of all cells to express the positive markers was rarely achieved. Only cells derived from synovium from the early OA patients expressed CD73 by >95% (means: synovium, 95.8%, bone, 93.4%). Both cell types from non-OA patients showed lower expression of CD73 (means: synovium, 86.9%, bone, 91.4%) (Figure 11a). However, when adjusted for the age, the difference was not statistically significant for neither synovium nor bone-derived cells between both groups of patients (general linear model with Bonferroni post hoc). Even lower percentage of the cells expressed CD90 with no differences between early OA and non-OA patients (means: synovium, 92.5%; bone, 92.9%; and synovium, 79.1%; bone, 91.5%, respectively). The lowest percentage of the cells expressed CD105 with no differences between early OA and non-OA patients (means: synovium, 78.5%; bone, 69.3%; and synovium, 86.7%; bone, 77.0%, respectively).

Then, for the negative markers, the ISCT criteria indicate that <2% of the cells should express these markers [40]. Here, the cells from both of the tissues for both early OA and non-OA patients fulfilled these criteria (means: synovium, 0.82%; bone, 0.46%; and synovium, 0.19%; bone, 1.3%, respectively) (Figure 11a). However, when adjusted for age, the difference was not statistically significant between both groups of patients (general linear model with Bonferroni post hoc).

### 3.6. Gene Expression Profiling

#### 3.6.1. Clusters of Cells from Non-OA Patients Express Similar Profiles of PDPN and CD146 Markers to Human Skeletal Stem Cells

**Figure 12:**
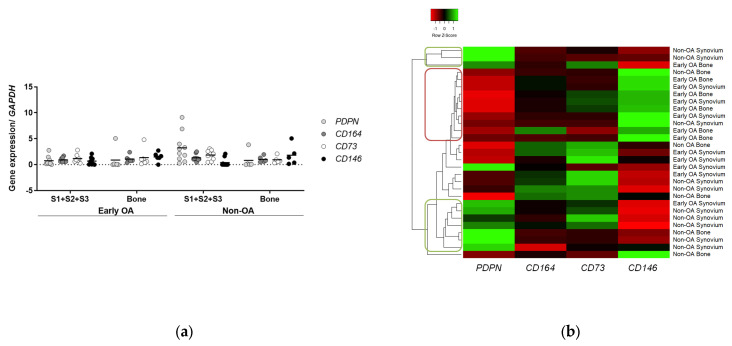
Skeletal stem cell markers gene expression profiling. (**a**) The gene expression of markers of skeletal stem cells was measured during in vitro expansion of the cells at passage 3. No significant difference was obtained for the four markers between synovium and bone-derived cells from early OA and non-OA patients (general linear model with Bonferroni post hoc). Individual samples and means are shown. S1 + S2 + S3 presents a group of combined data from S1, paralabral synovium, S2, cotyloid fossa and S3, peripheral inner surface of the joint capsule. (**b**) Hierarchical clustering of skeletal stem cell marker gene expression in all types of cells (rows, individual samples; columns, marker genes; red, gene expression lower than reference channel; green, gene expression higher than reference channel). The clustering tree analysis is also shown (left). Based on *PDPN* and *CD146* expression 2 clusters were identified. Red circle shows a cluster of 10 samples of low *PDPN* and high *CD146* expression with the majority (8) of early OA samples. Two green circles show 10 samples divided into 2 clusters of high *PDPN* and low *CD146* expression with the majority (8) of samples from non-OA patients.

Gene expression profiling was also performed for the four markers to determine whether the phenotype of human skeletal stem cells (i.e., PDPN/CD73/CD164 positive, CD146 negative) [13,14] also applies to these MSCs, and to distinguish the cells between the two groups of patients (Figure 12). The data for the multitest comparisons showed no differences in the expression of these genes between the cells from these two patient groups (general linear model with Bonferroni post hoc).

However, hierarchical clustering of the expression of these four marker genes identified a small cluster of 10 of these cell samples with low *PDPN* and high *CD146* expression (Figure 12b, red circle), with the majority of these being the cells from the early OA patients (8 of 10). In contrast, 2 separate clusters of 10 of these MSC samples showed high *PDPN* and low *CD146* expression, as reminiscent of human skeletal stem cells (Figure 12b, green circles), which consisted of 8 from the non-OA patients, and 2 from the early OA patients.

#### 3.6.2. Inflammatory and Immunomodulatory Markers

**Figure 13:**
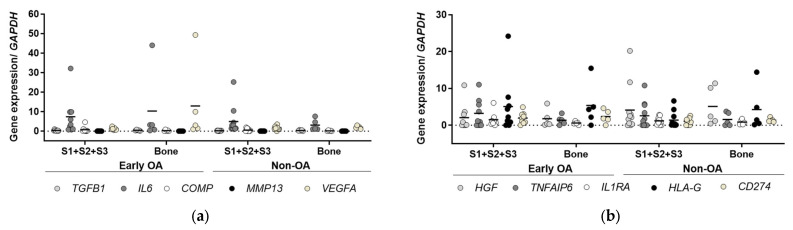
Inflammatory and immunomodulatory markers gene expression profiling. (**a**) The gene expression of inflammatory markers was measured during in vitro expansion of the cells at passage 3. No significant differences were observed for any of the markers between synovium and bone-derived cells from early OA and non-OA patients (general linear model with Bonferroni post hoc). (**b**) The gene expression of immunomodulatory markers was measured during in vitro expansion of the cells at passage 3. No significant difference was obtained in any of the markers between synovium and bone-derived cells from early OA and non-OA patients (general linear model with Bonferroni post hoc). Individual samples and means are shown. S1 + S2 + S3 presents a group of combined data from S1, paralabral synovium, S2, cotyloid fossa and S3, peripheral inner surface of the joint capsule.

Gene expression profiling was also performed for the five inflammatory (Figure 13a) and five immunomodulatory markers (Figure 13b) to distinguish the synovium and bone-derived cells between the two groups of patients. However, no differences were observed (general linear model with Bonferroni post hoc).

## 4. Discussion

In the present study, we show that MSCs derived from the synovium, namely the paralabral synovium, the cotyloid fossa, and the peripheral inner surface of the joint capsule, and also from bone, from patients with early OA have comparable biological properties to these cells from non-OA patients. Our study suggests that synovium-derived and bone-derived MSCs are not affected by the early degenerative changes in the hip. Moreover, these different anatomical sites of the human synovium represent alternative tissue sources to bone, and the derived cells have equivalent sought-after features of MSCs for patient-derived stem cell therapies, and they can be harvested during hip arthroscopy. In addition, we show that the combination of *PDPN* and *CD146* at the gene expression level can be used to distinguish MSCs between early OA and non-OA patients. A low *PDPN*/high *CD146* profile was mostly observed for these cells from the early OA patients, while those from the non-OA patients most commonly expressed the high *PDPN*/low *CD146* profile similar to skeletal stem cells [13,14].

Joint tissues have been studied as sources of MSCs that show different in vitro biological regenerative potential [1,5,12,25,41]. However, in orthopedics’ clinical practice, autologous bone marrow and adipose tissue are currently the most commonly used tissue sources of MSCs [4]. Such stem cell therapies have been shown to provide promising improvements for bone–joint injuries (e.g., fractures, bone defects, non-union, spinal injuries), OA–cartilage defects, ligament–tendon injuries, femoral–head osteonecrosis, and osteogenesis imperfecta, despite the lack of detailed scientific understanding of their mechanisms [2,3,15,17,18]. Considering that these stem cell therapies are derived from the patients themselves when they show clinical signs of degenerative joint disorders such as OA, there is a lack of knowledge of whether the MSCs used for such cell therapies are also affected by the concomitant disorder [5,6,7,8,9,12,42,43]. Our previous results have shown the exhaustion of the bone-derived MSCs in patients with primary OA. These cells have shown reduced viability, osteogenic and chondrogenic potential in vitro in comparison with their counterparts harvested from patients with OA as a consequence of hip dysplasia [12]. In addition to the age of the donor that has been shown to affect the properties of primary cells [9,44], the concomitant disorder must be taken into account when selecting the donor and tissue source for cell therapies.

There are numerous studies that have evaluated tissue-specific MSCs, most commonly bone-marrow-derived MSCs [5,6,7,8,12,19,23,24,25,28,32,33,41,45,46,47]. However, studies that have directly compared MSCs from different joint tissues within individual patients are rare [5,7,12,19,25,28,32], and except for advanced OA, not much is known about the impact of degenerative changes in the joints during the early stages of MSCs resident in different tissues. To this end, we performed detailed in vitro comparisons of primary cells derived from three synovial sites and from trabecular bone from patients with early hip OA (early OA) and patients with no such hip degeneration (non-OA).

Most of the in vitro studies to date that have investigated human MSCs have followed the criteria defined by the ISCT [40]. Following these criteria, the cells must be plastic adherent, positive for CD90, CD105 and CD73, and negative for CD45, CD34, CD14, CD19 and MHC-II, and they must undergo trilineage differentiation (i.e., adipogenic, osteogenic, chondrogenic). However, several studies have shown that the positive markers, in particular CD105, are not being expressed by MSCs as high as recommended by ISCT [48]. A significant step forward in human stem cell biology has been made by the discovery of self-renewing, multipotent and cartilage-regenerating human skeletal stem cells identified as podoplanin (PDPN), CD164 and CD73 positive, and CD146 negative [13,14]. In vitro analysis in the present study followed the ISCT recommendations to show that the primary cells derived from all four tissues here have MSC-like features. In addition, we also screened these cells for expression of the newly identified skeletal stem cell marker genes [13,14] and also tested their expression for common immune-related molecules to assess their immunomodulatory potential.

The present study can be compared to a recent study by Murata et al. [19] that investigated MSCs harvested from the paralabral synovium and cotyloid fossa from 18 donors during arthroscopic hip surgery for femoroacetabular impingement syndrome. Using standard in vitro assays, such as CFU, viability, and trilineage differentiation, they showed that MSCs from the cotyloid fossa have greater proliferation and differentiation potential than those from the paralabral synovium. They concluded that the cotyloid fossa in these patients appears to be a more promising tissue source for cell-based therapies for cartilage damage. In the present study, we did not see any significant differences between MSCs derived from synovium, i.e., paralabral synovium, cotyloid fossa and peripheral inner surface of the joint capsule in comparison with the most well-recognized source of MSC, the bone tissue. Of note, there remained no differences here also if we combined the two groups of patients (i.e., early OA plus non-OA; N = 16). It is reasonable to believe, therefore, that the discrepancy between these data and those of Murata et al. [19] will be due to the characteristics within the different patient groups analyzed. In the present study, for example, only one patient had femoroacetabular impingement syndrome (in the non-OA group), while in the study of Murata et al., all of the patients had symptomatic femoroacetabular impingement syndrome, and those with OA were excluded. Previously, Kohno et al. also showed no differences in biological properties such as yields, surface markers and chondrogenic potential of primary synovium MSCs in patients with rheumatoid arthritis in comparison with those with OA [23]. They concluded that the synovium derived from patients with rheumatoid arthritis is a suitable cell source of MSCs for cartilage and meniscus regeneration. In addition to an analysis of different patients, such as those with advanced OA and rheumatoid arthritis undergoing arthroplasty, they also harvested synovium from the bone side in the suprapatellar pouch of the knee. Hence, it is difficult to compare these two studies. Interestingly, similar to previous studies [7,13,19] we also found broad donor-to-donor variability in primary cell properties such as CFU-F, cumulative population doublings, differentiation potential and immunophenotype in particular in early OA patients. This might be the consequence of the degenerative changes occurring in the hip on MSCs.

Given the lack of clear characterization of MSCs in autologous cell concentrates derived from patients suffering from degenerative joint disorders, better identification of the features and potential of MSCs from different tissues remains of the utmost importance. Several previous studies have suggested that MSCs might be affected in some ways in patients with OA, especially considering bone-marrow-derived and cartilage-derived cells [6,7,9,12,42]. Hence, the use of these autologous tissues for the purpose of regenerative medicine remains questionable, and alternative sources are still needed. Synovium is a promising alternative source, and in particular, synovium MSCs have been shown to have greater potential for chondrogenesis both in vitro and also in vivo [1,20,21,22,25,26,41,49,50]. However, first, it needs to be demonstrated that different tissue-derived MSCs have such preferable properties using detailed and robust in vitro analyses.

Thus, in addition to standard in vitro tests that would have paralleled previous studies on MSCs, we also performed gene expression profiling for the recently discovered markers of bone-marrow-resident human skeletal stem cells [13,14]. Through this, we identified a cluster of MSCs that showed high *PDPN* and low *CD146* expression, reminiscent of human skeletal stem cells. This cluster encompasses mainly cells from the non-OA patients, while the cells from early OA patients mostly clustered as low *PDPN* and high *CD146*. Murphy et al. recently showed that these stem cells decrease with age and possibly to development of OA [13]. However, following microfracture and a local application of BMP2 and a soluble antagonist of VEGFR1, these stem cells could be induced to regenerate cartilage. In line with this finding, our results suggest the absence of these types of stem cells, i.e., high *PDPN*/low *CD146* is related to degenerative changes in the hip such as those observed in our patients with early OA. We have previously compared the skeletal stem cell markers between early and late OA patients and non-OA (*post mortem* donors) on gene and protein levels [33]. These results showed high *CD146* and low gene expression of the three positive markers in late OA patients, which is in accordance with the current study. However, the immunophenotyping in our previous study identified a lower percentage of CD164 cells in early OA patients in comparison with late OA patients and no OA donors [33]. The results of the current study were obtained on gene expression level only, hence further immunophenotyping of these cells is required to compare the results of both studies.

Taken together, the significance and novelty of our findings are seen in the direct comparison of the in vitro biological features of synovium-derived and bone-derived MSCs in early OA patients and non-OA patients. Previous studies that have investigated the in vitro potential of tissue-specific MSCs from various donors included a narrow age range for the patients or did not definitively identify donors with OA versus healthy patients across a wide range of advancing ages. Here, instead, we have shown that the synovium and bone in patients with early degenerative changes can provide MSCs that show comparable in vitro properties to those of patients with no degenerative changes. Furthermore, we have provided evidence that synovium and bone represent sources of MSCs that are not affected by early hip degeneration. The biological features tested in our study are key properties for the selection of optimal cell sources for cellular therapies.

The main drawback of the present study is that we were not able to perform all of the analyses for all of the tissue samples. This is because the expansion of primary cell cultures can be a difficult procedure, and not all cell samples will expand enough beyond p0 to provide sufficient quantities of cells for all of the experiments carried out, and, in particular, for the differentiation studies. In addition, the amounts of tissue that can harvested during arthroscopy are relatively small in comparison with samples of bone marrow from the iliac crest or abdominal fat. This presents also a major limitation for the clinical application of synovium-derived cell therapies. However, this is a genuine problem, and for the patient tissue samples that provided cells, is comparable to previous studies [7,19,23].

To identify the optimal tissue for the purpose of regenerative medicine in patients with different types of OA, further comparative studies are required. These will need to provide sufficient quantities of tissue harvested by minimally invasive procedures, and they need to be accompanied by high success rates for primary cell isolation.

## 5. Conclusions

In conclusion, we have shown here that synovium and bone in patients with early hip OA represent sources of MSCs that have comparable in vitro biological properties to those from patients with no degenerative changes in the hip. Moreover, for patient-derived stem cell therapies, synovium represents an alternative tissue source of MSCs that are comparable to those from bone. Finally, we also identified a cluster of mainly non-OA MSCs that show the high *PDPN*/low *CD146* profile that is reminiscent of skeletal stem cells. These findings should also encourage further studies, in particular in in vivo animal OA models, to investigate the potential of PDPN and CD146 as biomarkers in joint degeneration.

## Figures and Tables

**Table 1:** Patient clinical characteristics and arthroscopic findings.

No.	Patient Group	Age (years)	Sex (M/F)	BMI (kg/m^2^)	Side (L/R)	Diagnosis	Treatment
1	Early OA	36	F	21.3	L	Degenerative labrum changes with rupture	Labrum resection, acetabuloplasty
5		46	F	24.6	L	Labrum lesion	Labrum resection
6		47	F	22.4	R	Degenerative labrum changes with rupture	Labrum resection, acetabuloplasty
8		60	M	28.9	L	Degenerative labrum changes with rupture	Labrum resection, acetabuloplasty
11		49	M	26.2	R	Degenerative labrum changes with rupture	Labrum refixation, minimal acetabuloplasty, femoral head-neck osteoplasty
12		54	F	29.7	L	Degenerative labrum changes with rupture, pincer impingement	Labrum resection, acetabuloplasty
14		45	F	23.6	R	Early degenerative changes	Labrum refixation
16		48	F	24.8	L	Early degenerative changes	Labrum refixation
2	Non-OA	44	F	22.7	L	Labrum lesion	Labrum refixation
3		18	M	23.0	R	Mild acetabular dysplasia, hypertrophic labrum changes with rupture	Labrum refixation
4		15	F	18.8	R	Mild acetabular dysplasia	Diagnostic arthroscopy
7		42	M	23.5	L	Femoroacetabular impingement syndrome	Labrum refixation, femoral head-neck osteoplasty
9		45	F	25.0	R	Revision after arthroscopic labrum refixation	Resection of adhesions
10		24	F	22.1	L	Suspected labrum lesion	Diagnostic arthroscopy
13		23	F	25.6	L	Labrum lesion	Diagnostic arthroscopy
15		23	F	20.0	L	Labrum lesion	Labrum refixation

BMI, body mass index; M, male; F, female; OA, osteoarthritis; L/R, left/right.

## Data Availability

The data presented in this study are available on request from the corresponding author. The data are not publicly available due to restrictions, such as donor privacy protection, and ethical considerations.

## Supplementary Materials

The following supporting information can be downloaded at: https://www.mdpi.com/article/10.3390/cells13151238/s1, Figure S1: The representative images of the colonies formed at p0 are shown. S1, paralabral synovium, S2, cotyloid fossa and S3, peripheral inner surface of the joint capsule. Scale bars: 1000 µm
