# Supplementary material for: Synovium-Derived and Bone-Derived Mesenchymal Stem/Stromal Cells from Early OA Patients Show Comparable In Vitro Properties to Those of Non-OA Patients
Source: Cells. 2024 Jul 23;13(15):1238. doi: 10.3390/cells13151238 (PMC11311703; doi:10.3390/cells13151238)
Supplement: Supplementary file 1 [file cells-13-01238-s001.zip › cells-3084521-supplementary.pdf]

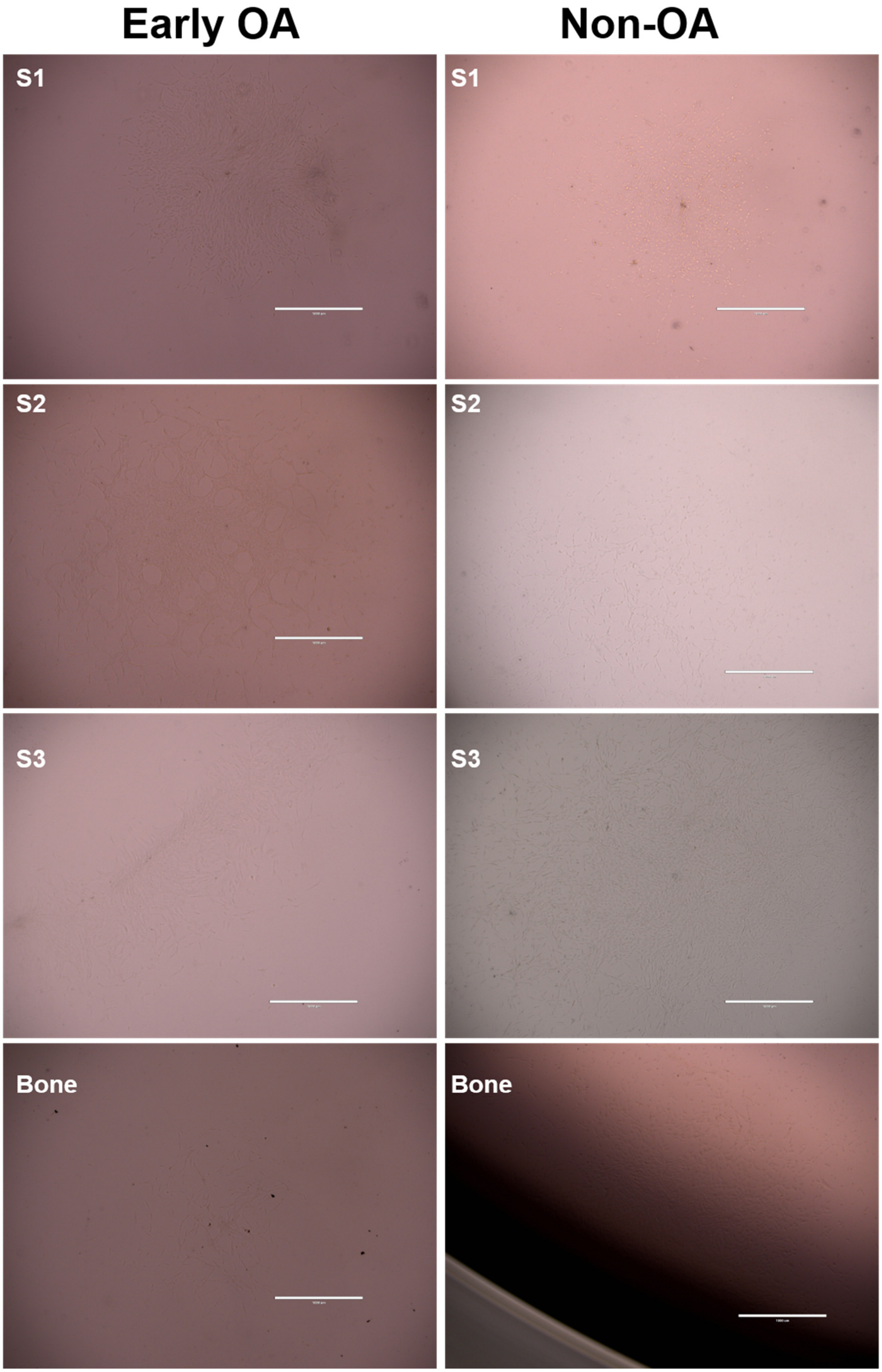

Figure S1: The representative images of the colonies formed at p0 are shown. .. S1, paralabral synovium, S2, cotyloid fossa and S3, peripheral inner surface of the joint capsule. Scale bars: 1000 µm
